# One-Pot Synthesis of Alumina-Titanium Diboride Composite Powder at Low Temperature

**DOI:** 10.3390/ma14164742

**Published:** 2021-08-22

**Authors:** Xueyin Liu, Ke Bao, Junfeng Chen, Quanli Jia, Shaowei Zhang

**Affiliations:** 1 College of Civil Engineering and Architecture, Quzhou University, Quzhou 324000, China; kklldliu@163.com; 2 College of Engineering, Mathematics and Physical Sciences, University of Exeter, Exeter EX4 4QF, UK; kb357@exeter.ac.uk; 3 The State Key Laboratory of Refractories and Metallurgy, Wuhan University of Science and Technology, Wuhan 430081, China; chenjunfengref@163.com; 4 Henan Key Laboratory of High Temperature Functional Ceramics, Zhengzhou University, Zhengzhou 450052, China; jiaquanli@zzu.edu.cn

**Keywords:** alumina, titanium diboride, composite powder, molten salt synthesis, aluminothermic reduction, boron oxide, titania, thermodynamic calculation

## Abstract

Alumina-titanium diboride (Al_2_O_3_-TiB_2_) composite powders were synthesised via aluminothermic reduction of TiO_2_ and B_2_O_3_, mediated by a molten chloride salt (NaCl, KCl, or MgCl_2_). The effects of salt type, initial batch composition, and firing temperature/time on the phase formation and overall reaction extent were examined. Based on the results and equilibrium thermodynamic calculations, the mechanisms underpinning the reaction/synthesis processes were clarified. Given their evaporation losses at test temperatures, appropriately excessive amounts of Al and B_2_O_3_ are needed to complete the synthesis reaction. Following this, phase-pure Al_2_O_3_-TiB_2_ composite powders composed of 0.3–0.6 μm Al_2_O_3_ and 30–60 nm TiB_2_ particles were successfully fabricated in NaCl after 5 h at 1050 °C. By increasing the firing temperature to 1150 °C, the time required to complete the synthesis reaction could be reduced to 4 h, although the sizes of Al_2_O_3_ and TiB_2_ particles in the resultant phase pure composite powder increased slightly to 1–2 μm and 100–200 nm, respectively.

## 1. Introduction

Alumina (Al_2_O_3_) is a representative high performance ceramic material possessing numerous superior properties. Apart from its high melting point, it exhibits high hardness and mechanical strength, excellent wear and chemical resistances, as well as good electrical/thermal insulation [1]. These, along with its ready availability, make Al_2_O_3_ an attractive ceramic material applicable to a wide range of structural and functional applications, in, e.g., wear-resistant components, high-speed cutting tools, armors, refractory linings/crucibles, electrical and chemical insulators, and artificial hip joints [2,3,4]. Unfortunately, it suffers from low fracture toughness and poor thermal shock resistance [5,6], negatively affecting its service performance and life. One of the commonly adopted strategies for overcoming this is to make a composite [7], by combining it with another reinforcement phase, e.g., particles/platelets, or fibrous phases such as carbon fibres and carbon nanotubes. Titanium diboride (TiB_2_) stood out as one of the reinforcement phases, owing to its good structural and thermodynamic compatibility with Al_2_O_3_ [8,9] and its excellent properties such as high melting point, high hardness/elastic modulus, relatively low density, and good thermal/electrical conductivities [10]. Previous studies found that incorporation of TiB_2_ particles into Al_2_O_3_ conferred much improved hardness, strength, fracture toughness and electrical conductivity on the resultant Al_2_O_3_-TiB_2_ composite materials [11,12,13,14,15,16], making them suitable for a variety of demanding applications, e.g., in electrodes, cutting tools, wear parts, lightweight armors, high-temperature/glow-plug heaters, and heat exchangers [14,17,18].

To fabricate high performance Al_2_O_3_-TiB_2_ composites, it is key to prepare and use high quality counterpart powders. Conventionally, such composite powders were prepared by directly mixing pre-synthesised TiB_2_ and Al_2_O_3_ powders in a ball mill [11,12,14,15,16,17,19,20]. Unfortunately, the final bulk composites exhibited unsatisfactory service performance arising mainly from poor dispersion and distribution of TiB_2_ in Al_2_O_3_. To overcome this, in-situ formation of the two constituent phases was suggested [21]. For example, a self-propagating high-temperature synthesis (SHS) method was attempted to form in-situ Al_2_O_3_ and TiB_2_ from powder mixtures of Al, TiO_2_/Ti, and B_2_O_3_/B/H_3_BO_3_ [18,22,23,24,25,26,27,28,29,30,31,32]. However, this method suffered from several weaknesses; in particular, difficulties to control the synthesis process and synthesise phase pure materials. Apart from SHS, mechanochemical synthesis [8,9,33,34,35], and milling assisted sol-gel process [36] were investigated. With these two techniques, a long processing time was required, and the resultant powders were often heavily agglomerated, and contaminated during prolonged milling.

In recent years, our group has successfully synthesised several ultra-high temperature boride powders via magnesiothermic reduction of oxide precursors in a molten salt [37,38,39]. In this work, such a molten salt synthesis (MSS) technique was further developed to synthesise high-quality Al_2_O_3_-TiB_2_ composite powders at relatively low temperatures. The effects of processing factors such as salt type, initial batch composition, and firing temperature/time on the synthesis process were examined, and the synthesis conditions optimised. Based on the results, and thermodynamic calculations, the mechanisms underpinning the reaction/synthesis process were proposed.

## 2. Materials and Methods

The TiO_2_ (≥99%, 100–300 nm), B_2_O_3_ (99.98%) and Al (99.5%, ~44 μm) powders were used as the main starting materials, along with KCl (>99%), NaCl (>99%) and anhydrous MgCl_2_ (>98%). All of these materials except for Al (Alfa Aesar, Lancashire, UK) were from Sigma-Aldrich (Gillingham, UK). The three main starting materials were mixed in stoichiometric (indicated by the overall Reaction (1)) or nonstoichiometric proportions (with excess Al and B_2_O_3_), in a mortar and pestle, and further combined with 5 times (by weight) of each of the salts. The final powder mix was contained in an alumina crucible and heated in an argon protected tube furnace to a temperature between 850 and 1150 °C and held for 4–6 h. After cooling to room temperature, the reacted powder coexisting with the residual salt was subjected to repeated hot water washing. The remaining salt-free product powder was dried overnight at 80 °C before being subjected to detailed characterisations.

3TiO_2_ + 3B_2_O_3_ + 10Al = 3TiB_2_ + 5Al_2_O_3_ (1)

Phase formation and reaction extent in samples were evaluated based on their X-ray diffraction (XRD) spectra recorded by an X-ray diffractometer (D8 Advance, Bruker, Germany) operated at 40 mA/40 kV (using Cu*K*α radiation), and at a scan rate of 2° (2θ) min^−1^, and the ICDD cards used are: TiB_2_ (35-741), Al_2_O_3_ (corundum) (10-173), Ti_2_O_3_ (43-1033), Al_3_Ti (37-1449), Al_18_B_4_O_33_ (32-3), and MgAl_2_O_4_ (21-1152). Samples were also gold- or carbon-coated for further microstructural examinations using a scanning electron microscope (SEM, Nova 600, FEI, Hillsboro, OR, USA) and a transmission electron microscope (TEM, JEM 2100, JEOL, Tokyo, Japan), linked with energy-dispersive X-ray spectroscopy (EDS, Oxford Instrument, Oxford, UK).

To assist in clarification of the mechanisms underpinning the whole reaction/synthesis process, thermodynamic calculations were carried out using the commercial FactSage package [40]. The Gibbs energy values for each of the intermediate reactions and the overall reaction (Reaction (1)), as a function of temperature, were calculated.

## 3. Results and Preliminary Discussion

### 3.1. Influence of Salt Type on Al_2_O_3_-TiB_2_ Formation

**Figure 1:**
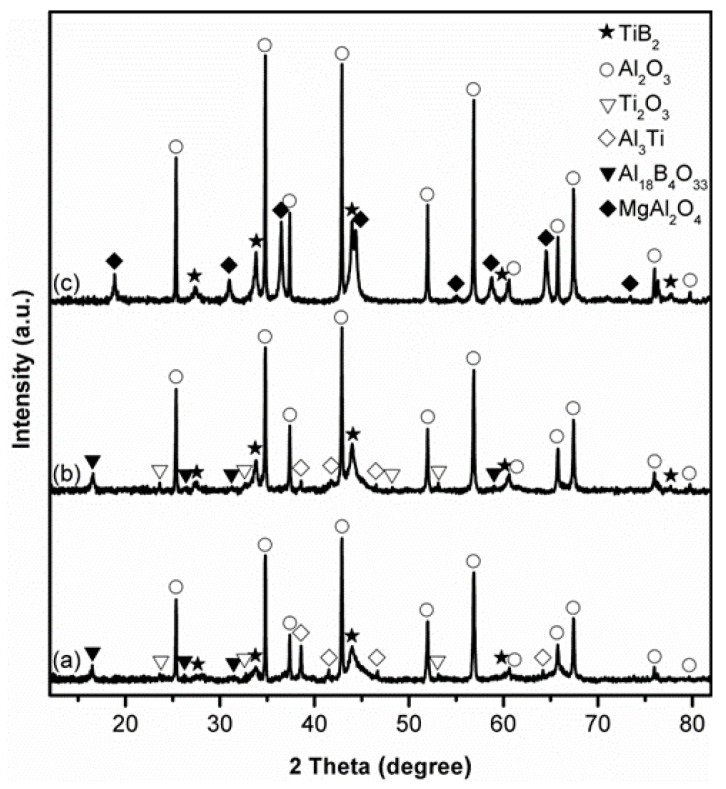
XRD patterns of stoichiometric samples after 4 h firing at 850 °C in: (**a**) KCl, (**b**) NaCl, and (**c**) MgCl_2_.

Figure 1 shows XRD patterns of stoichiometric samples after 4 h firing in different salts at 850 °C. After firing in KCl, α-Al_2_O_3_ and TiB_2_ were identified as the primary phases in the sample (Figure 1a). Some intermediate Al_18_B_4_O_33_ and Al_3_Ti were also detected, along with a trace of Ti_2_O_3_, indicating the low extents of Reaction (1) and Al_2_O_3_-TiB_2_ formation. Replacing KCl with NaCl (Figure 1b) led to an evident increase in the relative peak heights of both Al_2_O_3_ and TiB_2_, and a decrease in those of Al_3_Ti, while those of intermediate Al_18_B_4_O_33_ and Ti_2_O_3_ changed little, demonstrating the enhanced extents of Al_2_O_3_-TiB_2_ formation, and the better effect of NaCl than KCl on accelerating the overall reaction. As discussed previously [37,38,39], several processing parameters—in particular, the solubility and mobility of reaction species in the molten salt—play crucial roles in MSS. Unfortunately, these key processing parameters are not available, so it is difficult to figure out the reasons behind the better accelerating effect of NaCl in the present case. When NaCl was replaced with MgCl_2_, the peak heights of Al_2_O_3_ and TiB_2_ further increased (Figure 1c), and no Al_18_B_4_O_33_, Al_3_Ti or Ti_2_O_3_ was detected, suggesting much enhanced extents of Reaction (1). Nevertheless, an undesirable by-product phase, magnesium aluminate spinel (MgAl_2_O_4_), was formed in this case, due to likely the reaction between the formed Al_2_O_3_ and the MgCl_2_ (Reaction (2) [41]. In the three cases, except for α-Al_2_O_3_, no other transition aluminas were detected at such a low firing temperature. The reason for this was not clear, but it could be due to the locally enhanced salt bath temperature arising from the exothermic reactions, as suggested by the thermodynamic calculations presented later (see Section 4 below). Given the better accelerating effect of NaCl than KCl, and the formation of undesired by-product MgAl_2_O_4_ in the case of using MgCl_2_, NaCl was chosen specifically to form a liquid reaction medium for the following MSS investigations.

4Al_2_O_3_ + 3MgCl_2_ = 3MgAl_2_O_4_ + 2AlCl_3_ (2)

### 3.2. Influence of Firing Temperature on Al_2_O_3_-TiB_2_ Formation

**Figure 2:**
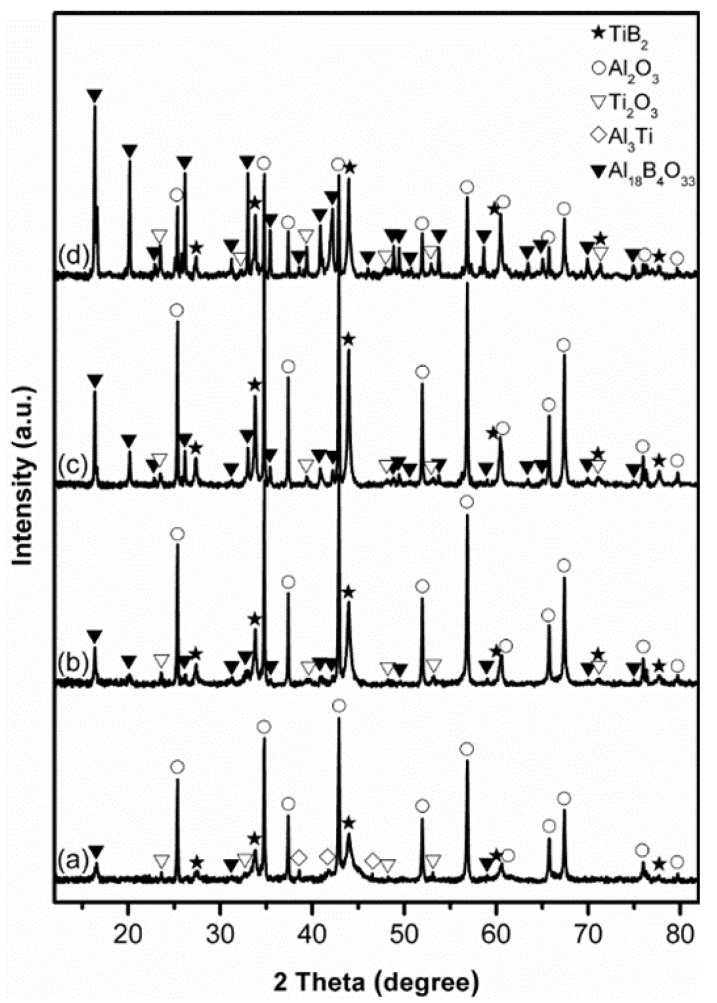
XRD patterns of stoichiometric samples after 4 h firing in NaCl at: (**a**) 850, (**b**) 950, (**c**) 1050, and (**d**) 1150 °C.

Figure 2 presents XRD patterns of stoichiometric samples after 4 h firing in NaCl at different temperatures. At 850 °C, as already described above, Al_2_O_3_ and TiB_2_ were formed as the primary phases, but some intermediate Al_18_B_4_O_33_, Al_3_Ti and Ti_2_O_3_ were detected (Figure 2a/Figure 1b). Upon increasing the temperature to 950 (Figure 2b) and 1050 °C (Figure 2c), the relative peak heights of Al_2_O_3_ and TiB_2_ increased significantly. Meanwhile, Al_3_Ti disappeared, and Ti_2_O_3_ changed little, but the peak heights of Al_18_B_4_O_33_ increased. These results implied that increasing the firing temperature favoured not only the Al_2_O_3_-TiB_2_ formation but also the Al_18_B_4_O_33_ formation. Further increasing the temperature to 1150 °C led to a further increase in the peak heights of Al_18_B_4_O_33_ and Ti_2_O_3_ but decrease in those of Al_2_O_3_ and TiB_2_ (Figure 2d), indicating the adverse effect of such further temperature increase on the Al_2_O_3_-TiB_2_ formation. This, according to our previous studies on MSS of transition metal borides [37,38,39], was due to probably evaporation loss of Al at relatively high reaction temperatures [42]. To confirm this, the influence of using excess Al on the Al_2_O_3_-TiB_2_ formation was further investigated, as described and discussed next.

### 3.3. Influence of Excess Al on Al_2_O_3_-TiB_2_ Formation

**Figure 3:**
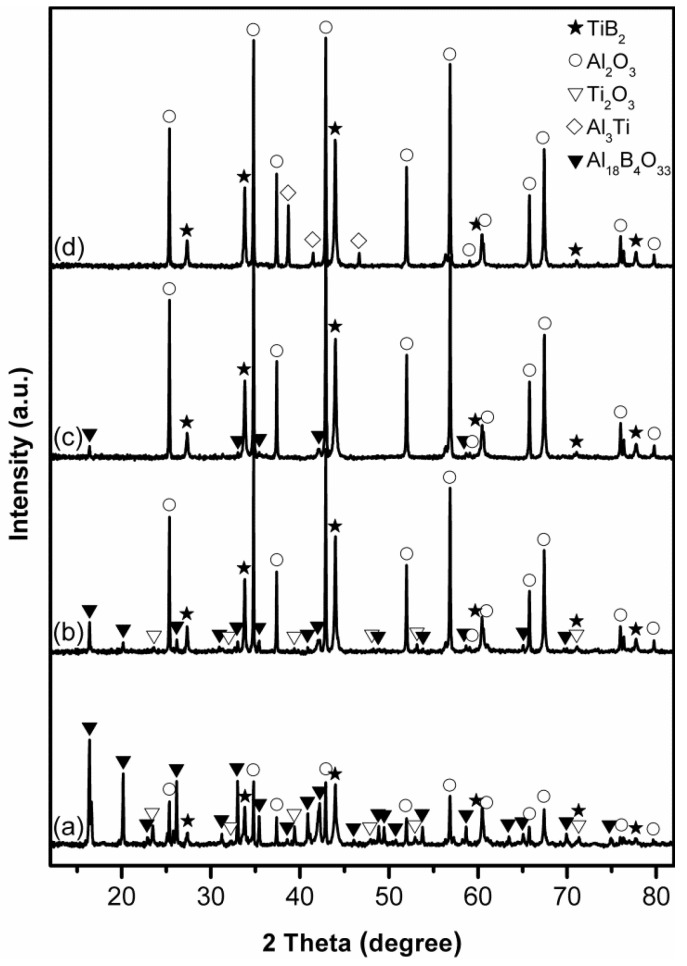
XRD patterns of samples using (**a**) 0, (**b**) 20, (**c**) 25, and (**d**) 30 wt% excess Al, after 4 h firing in NaCl at 1150 °C.

Figure 3 illustrates the influence of excess Al on phase evolution in samples after 4 h firing in NaCl at 1150 °C. The use of 20 wt% more Al resulted in considerable increase in the peak heights of Al_2_O_3_ and TiB_2_ and decrease in those of Al_18_B_4_O_33_ and Ti_2_O_3_ (Figure 3a,b). By further increasing excess Al to 25 wt%, the peak heights of Al_2_O_3_ and TiB_2_ were further increased. Meanwhile, Ti_2_O_3_ was completely eliminated, and only minor Al_18_B_4_O_33_ remained (Figure 3c), illustrating positive effects from the Al compensation. Nevertheless, the formation of phase-pure Al_2_O_3_-TiB_2_ was still not achieved.

To eliminate Al_18_B_4_O_33_, the excess Al was further increased to 30 wt%. Unfortunately, some Al_3_Ti was formed instead (Figure 3d), implying that there was no sufficient supply of B in the system, due to probably evaporation loss of B_2_O_3_ at such a relatively high temperature [43,44].

### 3.4. Influence of Excess B_2_O_3_ on Al_2_O_3_-TiB_2_ Formation

**Figure 4:**
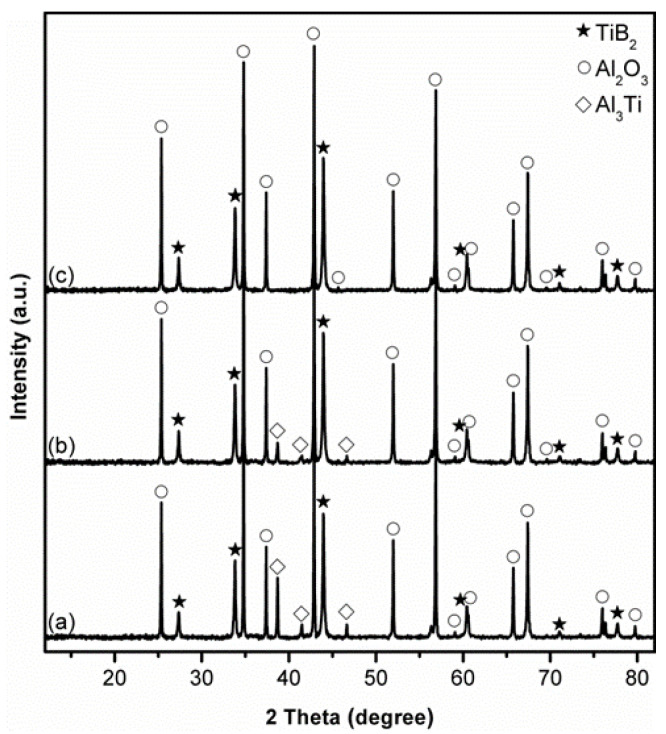
Influence of excess B_2_O_3_ on phase formation in samples using 30 wt% excess Al, after 4 h firing in NaCl at 1150 °C: (**a**) 0 (stoichiometric), (**b**) 10, and (**c**) 20 wt% excess B_2_O_3_.

To confirm and address the issue with B_2_O_3_ evaporation loss, the influence of using excess B_2_O_3_ (along with 30 wt% excess Al) on the Al_2_O_3_-B_2_O_3_ formation in the sample fired at 1150 °C for 4 h was further investigated as an example. When 10 wt% excess B_2_O_3_ was used, the peak heights of both Al_2_O_3_ and TiB_2_ increased, whereas those of Al_3_Ti decreased considerably (Figure 4b), compared with in the case of stoichiometric sample (Figure 4a). Further increasing the excess B_2_O_3_ to 20 wt% led to complete elimination of Al_3_Ti, and the formation of phase pure Al_2_O_3_-TiB_2_ (Figure 4c).

### 3.5. Influence of Firing Time on Al_2_O_3_-TiB_2_ Formation and Further Optimisation of Synthesis Condition

**Figure 5:**
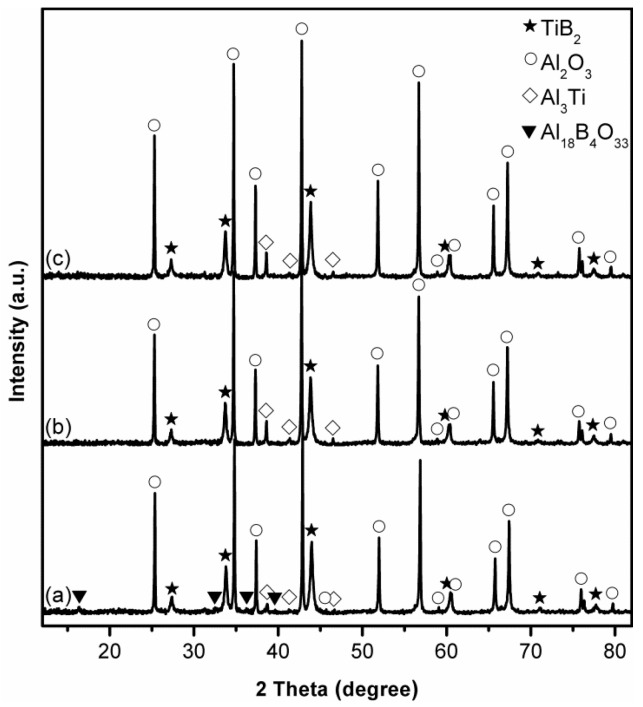
XRD patterns of samples using 30 wt% excess Al and 20 wt% excess B_2_O_3_ after firing in NaCl at 1050 °C for: (**a**) 4, (**b**) 5, and (**c**) 6 h.

**Figure 6:**
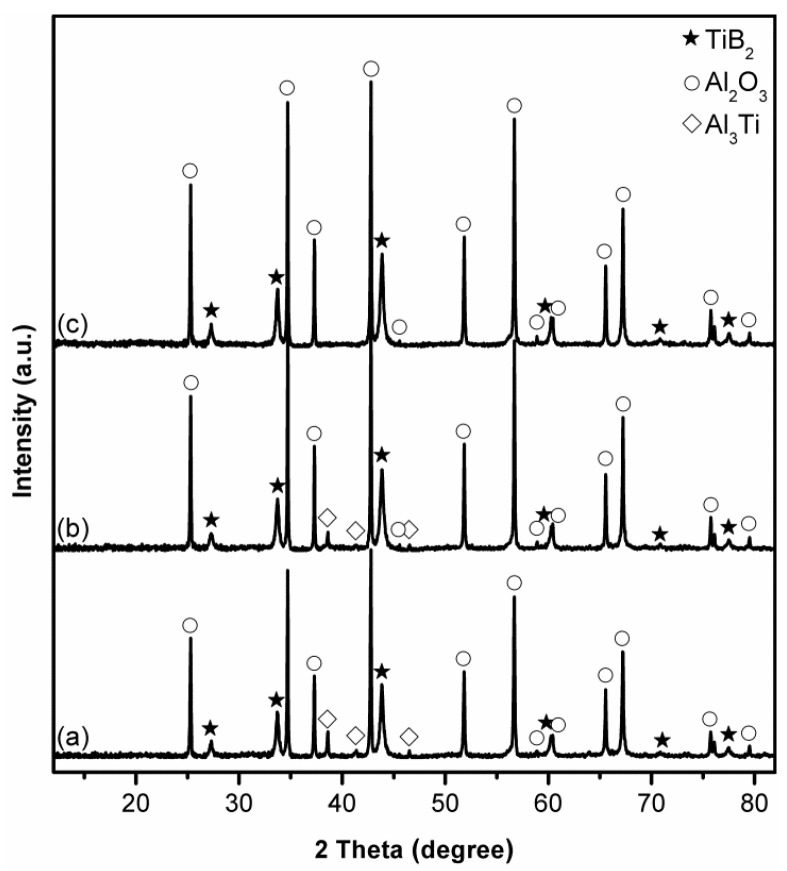
XRD patterns of samples after firing in NaCl at 1050 °C for 5 h using 30 wt% excess Al, and (**a**) 20, (**b**) 25, and (**c**) 30 wt% excess B_2_O_3_.

To reveal the influence of firing time on the Al_2_O_3_-TiB_2_ formation and further optimise the synthesis condition, samples containing 30 wt% excess Al and 20 wt% excess B_2_O_3_ were fired in NaCl at 1050 °C for different time periods, and then analysed by XRD (Figure 5). As shown in Figure 5a,b, with increasing the time at 1050 °C from 4 to 5 h, Al_18_B_4_O_33_ disappeared, whereas the peak heights of Al_3_Ti increased. Further extending the time to 6 h resulted in little change in the XRD pattern (Figure 5c), and Al_3_Ti was still detected. This, as mentioned above (Section 3.4), indicated that 20 wt% excess B_2_O_3_ might not be sufficient to compensate for the evaporation loss of B_2_O_3_ under this condition, in other words, more excess B_2_O_3_ had to be used. As verified by Figure 6, increasing excess B_2_O_3_ from 20 wt% to 25 wt% led to significant decrease in the peak heights of Al_3_Ti, and further increasing the excess amount to 30 wt%, all the intermediate phases including Al_3_Ti disappeared, and phase pure Al_2_O_3_-TiB_2_ powder was finally obtained.

### 3.6. Microstructure of Al_2_O_3_-TiB_2_ Product Powder

**Figure 7:**
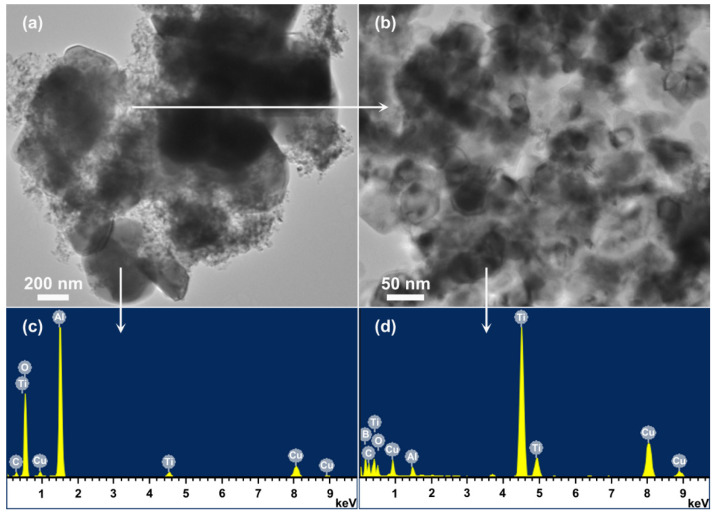
TEM images (**a**,**b**) and corresponding EDS (**c**,**d**) of the Al_2_O_3_-TiB_2_ composite powder resultant from 5 h firing in NaCl at 1050 °C (the small C and Cu peaks arose from the carbon coating and the Cu grid).

**Figure 8:**
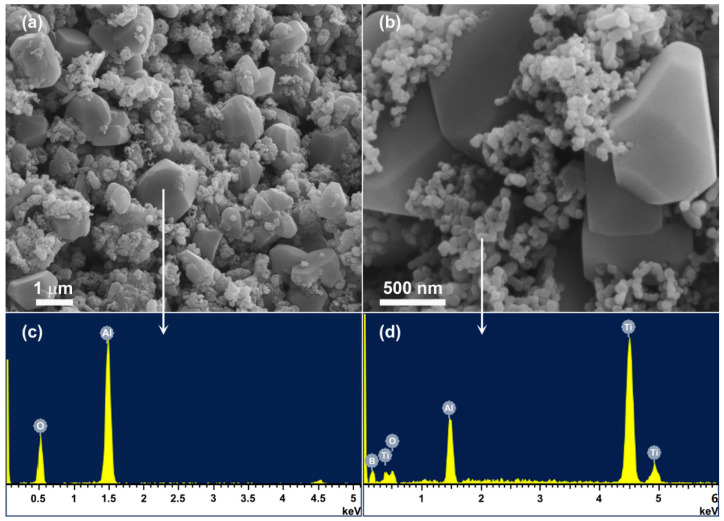
SEM images (**a**,**b**) and corresponding EDS (**c**,**d**) of the Al_2_O_3_-TiB_2_ composite powder resultant from 4 h firing in NaCl at 1150 °C.

As demonstrated in Figure 4c and Figure 6c and discussed above, phase pure Al_2_O_3_-TiB_2_ powder could be formed in NaCl after 5 h firing at 1050 °C (using 30 wt% excess Al and 30 wt% excess B_2_O_3_), or 4 h firing at 1150 °C (using 30 wt% excess Al and 20 wt% excess B_2_O_3_), which was further confirmed by microstructural characterisation. Figure 7, as an example, gives TEM images of the product powder prepared under the former condition, along with the corresponding EDS, showing the coexistence of submicron-sized Al_2_O_3_ particles (0.3–0.6 μm) and nanosized TiB_2_ particles (30–60 nm). Except these two phases, no other impurity phases were seen, as already revealed by XRD (Figure 6c). Figure 8 further displays SEM images of the product powder formed under the latter condition mentioned above. The formation of phase pure Al_2_O_3_-TiB_2_ in this case was also confirmed by EDS, along with XRD (Figure 4c). However, due to the higher synthesis temperature, Al_2_O_3_ and TiB_2_ particles in the composite powder became slightly bigger (1–2 μm Al_2_O_3_ and 100–200 nm TiB_2_).

## 4. Further Discussion and Reaction/Synthesis Mechanism

Based on the results presented in Figure 1, Figure 2, Figure 3, Figure 4, Figure 5, Figure 6, Figure 7 and Figure 8 and the preliminary discussion above, as well as on the thermodynamic calculations (Figure 9), the reaction/synthesis mechanisms (taking the case of using NaCl as an example) could be proposed; they are discussed as follows.

The firing temperatures (850–1150 °C) were above the melting points of NaCl, B_2_O_3_ and Al. So, at these temperatures, they all melted, forming a liquid NaCl pool, and B_2_O_3_ and Al liquid droplets. Then, the latter two would start to react with each other in the molten NaCl medium, forming B and Al_2_O_3_, according to Reaction (3).

On the other hand, as discussed in our previous work on MSS of TiB_2_ [39], TiO_2_ also would be reduced by the Al droplets in the molten NaCl medium, forming Ti and Al_2_O_3_ (Reaction (4)). The Al_2_O_3_ formed from Reactions (3) and (4) would partially react with the unreduced B_2_O_3_, in the NaCl medium, forming the intermediate Al_18_B_4_O_33_ (Reaction (5)), as detected by XRD (Figure 1, Figure 2 and Figure 3 and Figure 5).

B_2_O_3_ + 2Al = 2B + Al_2_O_3_ (3)

3TiO_2_ + 4Al = 3Ti + 2Al_2_O_3_ (4)

9Al_2_O_3_ + 2B_2_O_3_ = Al_18_B_4_O_33_ (5)

With the optimisation of synthesis condition, the intermediate Al_18_B_4_O_33_ could be further reduced by Al in the molten NaCl, forming additional B and Al_2_O_3_ according to Reaction (6), as verified by XRD (Figure 3 and Figure 5).

Apart from Al_18_B_4_O_33_, another main intermediate phase, Al_3_Ti, was formed from the reaction between the Ti formed from Reaction (4), and Al (Reaction (7)), as detected by XRD (Figure 1, Figure 2, Figure 3, Figure 4, Figure 5 and Figure 6).

As discussed, and confirmed in our previous studies [37,38,39], the formed B and Ti (Reactions (3), (4) and (6)) were slightly dissoluble in the molten chloride salts. So, the dissolved B and Ti would diffuse through the molten salt medium and reacted with each other, forming the other desirable phase of TiB_2_ (Reaction (8)).

Al_18_B_4_O_33_ + 4Al = 4B + 11Al_2_O_3_ (6)

Ti + 3Al = Al_3_Ti (7)

Ti + 2B = TiB_2_ (8)

B dissolved in the molten salt would also diffuse through the salt medium and react respectively with the Al_3_Ti produced from Reaction (7), and the unreacted raw materialTiO_2_, forming additional TiB_2_ (Reactions (9) and (10)). Reaction (9) was mainly responsible for the complete elimination of this intermediate phase under the optimal conditions (Figure 4 and Figure 6).

Al_3_Ti + 2B = TiB_2_ + 3Al (9)

10B + 3TiO_2_ = 3TiB_2_ + 2B_2_O_3_ (10)

**Figure 9:**
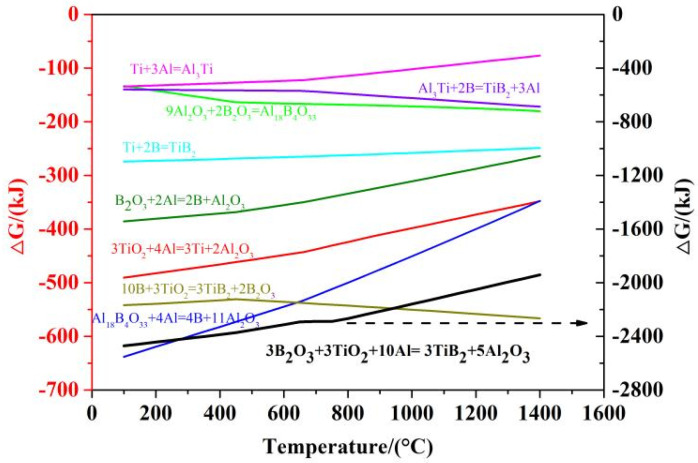
The standard Gibbs energy values corresponding to Reactions (1) and (3)–(10), as a function of temperature.

As shown in Figure 9, the Gibbs energy values corresponding to Reactions (3)–(9) and the overall Reaction (1) are also negative, at test temperatures (and even at lower temperatures), suggesting that all of these exothermic Reactions are thermodynamically favourable, which further supports the plausibility of the reaction/synthesis mechanisms proposed above.

## 5. Conclusions

Low temperature synthesis of Al_2_O_3_-TiB_2_ composite powders via aluminothermic reduction of TiO_2_ and B_2_O_3_ in a molten chloride salt (KCl, NaCl or MgCl_2_) was investigated. Processing parameters including salt type, initial batch composition, and firing temperature/time played important roles in the synthesis process. Phase pure Al_2_O_3_-TiB_2_ nanocomposite powder could be prepared in NaCl after 5 h firing at 1050 °C, when 30 wt% excess Al and 30 wt% excess B_2_O_3_ were used. The particle sizes of Al_2_O_3_ and TiB_2_ coexisting in the as-prepared composite powder were estimated to be 0.3–0.6 μm and 30–60 nm, respectively. By increasing the temperature to 1150 °C and using 30 wt% excess Al and 20 wt% excess B_2_O_3_, phase pure Al_2_O_3_-TiB_2_ composite powder also could be prepared in NaCl after only 4 h, although sizes of Al_2_O_3_ and TiB_2_ particles coexisting in the resultant composite power increased slightly to 1–2 μm and 100–200 nm, respectively.

## Data Availability

Data sharing is not applicable to this article.
